# Shifts in the Antibiotic Susceptibility, Serogroups, and Clonal Complexes of *Neisseria meningitidis* in Shanghai, China: A Time Trend Analysis of the Pre-Quinolone and Quinolone Eras

**DOI:** 10.1371/journal.pmed.1001838

**Published:** 2015-06-09

**Authors:** Mingliang Chen, Qinglan Guo, Ye Wang, Ying Zou, Gangyi Wang, Xi Zhang, Xiaogang Xu, Miao Zhao, Fupin Hu, Di Qu, Min Chen, Minggui Wang

**Affiliations:** 1 Key Laboratory of Medical Molecular Virology of Ministries of Education and Health, Institute of Medical Microbiology and Institutes of Biomedical Sciences, Fudan University, Shanghai, China; 2 Institute of Antibiotics, Huashan Hospital, Fudan University, Shanghai, China; 3 Shanghai Municipal Center for Disease Control and Prevention, Shanghai, China; 4 Intensive Care Unit, Shanghai Public Health Clinical Center Affiliated to Fudan University, Shanghai, China; Emory University, UNITED STATES

## Abstract

**Background:**

Fluoroquinolones have been used broadly since the end of the 1980s and have been recommended for *Neisseria meningitidis* prophylaxis since 2005 in China. The aim of this study was to determine whether and how *N*. *meningitidis* antimicrobial susceptibility, serogroup prevalence, and clonal complex (CC) prevalence shifted in association with the introduction and expanding use of quinolones in Shanghai, a region with a traditionally high incidence of invasive disease due to *N*. *meningitidis*.

**Methods and Findings:**

A total of 374 *N*. *meningitidis* isolates collected by the Shanghai Municipal Center for Disease Control and Prevention between 1965 and 2013 were studied. Shifts in the serogroups and CCs were observed, from predominantly serogroup A CC5 (84%) in 1965–1973 to serogroup A CC1 (58%) in 1974–1985, then to serogroup C or B CC4821 (62%) in 2005–2013. The rates of ciprofloxacin nonsusceptibility in *N*. *meningitidis* disease isolates increased from 0% in 1965–1985 to 84% (31/37) in 2005–2013 (*p* < 0.001). Among the ciprofloxacin-nonsusceptible isolates, 87% (27/31) were assigned to either CC4821 (n = 20) or CC5 (*n* = 7). The two predominant ciprofloxacin-resistant clones were designated China^CC4821-R1-C/B^ and China^CC5-R14-A^. The China^CC4821-R1-C/B^ clone acquired ciprofloxacin resistance by a point mutation, and was present in 52% (16/31) of the ciprofloxacin-nonsusceptible disease isolates. The China^CC5-R14-A^ clone acquired ciprofloxacin resistance by horizontal gene transfer, and was found in 23% (7/31) of the ciprofloxacin-nonsusceptible disease isolates. The ciprofloxacin nonsusceptibility rate was 47% (7/15) among isolates from asymptomatic carriers, and nonsusceptibility was associated with diverse multi-locus sequence typing profiles and pulsed-field gel electrophoresis patterns. As detected after 2005, ciprofloxacin-nonsusceptible strains were shared between some of the patients and their close contacts. A limitation of this study is that isolates from 1986–2004 were not available and that only a small sample of convenience isolates from 1965–1985 were available.

**Conclusions:**

The increasing prevalence of ciprofloxacin resistance since 2005 in Shanghai was associated with the spread of hypervirulent lineages CC4821 and CC5. Two resistant meningococcal clones China^CC4821-R1-C/B^ and China^CC5-R14-A^ have emerged in Shanghai during the quinolone era. Ciprofloxacin should be utilized with caution for the chemoprophylaxis of *N*. *meningitidis* in China.

## Abstract 2

Minggui Wang and colleagues assess the genetic origin and changing prevalence of *N*. *meningitidis* resistance to ciprofloxacin.

## Editors' Summary

**Background:**

Meningitis is a viral, bacterial, or fungal infection of the meninges, the thin membrane that covers the brain and the spinal cord. *Neisseria meningitidis* is the most common cause of bacterial meningitis in children and is a leading cause of meningitis in adults. *N*. *meningitidis* lives harmlessly in the mucous membranes of the nose and throat of 10%–20% of human beings. These symptom-free (asymptomatic) carriers are crucial to the transmission of *N*. *meningitidis*, which spreads from person to person through droplets of respiratory or throat secretions produced by infected individuals. Although *N*. *meningitidis* usually causes no harm, it occasionally overwhelms its host’s immune system and spreads through the bloodstream and into the brain. The characteristic symptoms of meningococcal meningitis—a rash, fever, stiff neck, sensitivity to light, confusion, headaches, and vomiting—then develop rapidly. Even with early treatment with intravenous antibiotics, about 10% of the 1.2 million people affected by meningococcal meningitis every year die, usually within 24–48 hours of symptom development.

**Why Was This Study Done?:**

Meningococcal meningitis can be prevented by vaccination against *N*. *meningitidis*. In addition, experts recommend that the family and close contacts of anyone with meningococcal meningitis be treated immediately with antibiotics to stop the disease spreading (*N*. *meningitidis* prophylaxis). The quinolone antibiotic ciprofloxacin is sometimes recommended for *N*. *meningitidis* prophylaxis, but quinolones have been used to treat many bacterial infections since the late 1980s and ciprofloxacin-resistant strains of *N*. *meningitidis* have recently emerged in some countries. Thus, policymakers in countries where ciprofloxacin may be used for meningococcal prophylaxis (for example, China, which has recommended several antibiotics, including ciprofloxacin, for meningococcal prophylaxis since 2005) need to know whether and how the susceptibility of *N*. *meningitidis* to ciprofloxacin is changing in their country. In this time trend analysis, the researchers investigate how the ciprofloxacin susceptibility, serogroup prevalence, and clonal complex prevalence of *N*. *meningitidis* have shifted in association with increasing use of quinolones in Shanghai (China), a region where many cases of meningococcal disease occur. A serogroup is a group of bacteria that carries a common antigen (a molecule recognized by the immune system); the prevalence of a serogroup is the proportion of the infected population that carries that serogroup. Only some *N*. *meningitidis* serogroups cause meningococcal disease. A clonal complex is a group of genetically related bacteria that may share genes that confer resistance to antibiotics. A strain of *N*. *meningitidis* is classified by both its serogoup and its clonal complex; the changing composition of prevalent strains can inform projections for disease spread and plans for disease management, including recommendations for prophylaxis.

**What Did the Researchers Do and Find?:**

The researchers analyzed the characteristics of 374 *N*. *meningitidis* isolates collected in Shanghai between 1965 and 2013 from patients with meningococcal disease, their close contacts, and asymptomatic *N*. *meningitidis* carriers identified in throat swab surveys. *N*. *meningitidis* serogroups and clonal complexes (CCs) shifted from predominantly serogroup A CC5 among isolates collected in 1965–1973, to serogroup A CC1 in 1974–1985, and to serogroup C or B CC4821 in 2005–2013. Notably, the rate of nonsusceptibility to ciprofloxacin in isolates from people with meningococcal meningitis increased from 0% in 1965–1985 to 84% in 2005–2013, and 87% (27/31) of the ciprofloxacin-nonsusceptible disease isolates belonged to either CC4821 or CC5. The researchers identified the two predominant ciprofloxacin-resistant strains (designated China^CC4821-R1-C/B^ and China^CC5-R14-A^) and showed that a different genetic alteration was responsible for the acquisition of antibiotic resistance in the two strains. Finally, the researchers report that, after 2005, the rate of ciprofloxacin nonsusceptibility among *N*. *meningitidis* isolates from asymptomatic carriers was 47%, and that some of the individuals with meningococcal meningitis shared nonsusceptible strains with their close contacts.

**What Do These Findings Mean?:**

These findings suggest that the increased prevalence of ciprofloxacin resistance seen in Shanghai since 2005 is associated with the spread of two hypervirulent clonal complexes of *N*. *meningitidis* (CC4821 and CC5) among individuals with meningococcal disease and within the healthy population. The findings also identify two resistant meningococcal strains (China^CC4821-R1-C/B^ and China^CC5-R14-A^) that have emerged in Shanghai since the use of quinolones to treat bacterial infections became widespread. Because this time trend analysis is based on a limited number of meningococcal isolates and because the researchers analyzed very few isolates collected between 1965 and 1985 and none collected between 1986 and 2004, this study cannot pinpoint exactly when, where, or how ciprofloxacin-resistant *N*. *meningitidis* emerged in Shanghai. Nevertheless, these findings suggest that the use of fluoroquinolones for *N*. *meningitidis* prophylaxis should be discouraged in China. Instead, the researchers suggest, physicians should turn to one of the other antibiotics recommended in China’s 2005 national scheme (for example, rifampicin) for the prevention and control of meningococcal meningitis.

**Additional Information:**

These websites can be accessed when viewing the PDF on a device, or via the online version of this article at http://dx.doi.org/10.1371/journal.pmed.1001838.

- The US Centers for Disease Control and Prevention provide information about meningococcal disease
- The World Health Organization has a fact sheet about meningococcal meningitis
- The UK National Health Service Choices website provides information about meningitis, including some personal stories
- The non-profit Meningitis Foundation of America also provides information about meningitis and stories from survivors of meningitis
- MedlinePlus has links to further resources about meningitis and about meningococcal infections; the MedlinePlus Encyclopedia also provides information about meningococcal meningitis (in English and Spanish)

## Introduction

*Neisseria meningitidis* is a leading cause of bacterial meningitis worldwide. At least 1.2 million cases of meningococcal disease are estimated to occur in the world annually, with approximately 10% resulting in death [1]. Based on variants in the capsular polysaccharide, 12 serogroups have been described, with the majority of invasive meningococcal disease caused by A, B, C, X, Y, or W [1]. The use of multi-locus sequence typing (MLST)—where seven housekeeping genes are sequenced to establish sequence types and clonal complexes (CCs)—has contributed substantially to our understanding of the molecular epidemiology of *N*. *meningitidis*, including isolates from different serogroups but with similar antigenic or disease phenotypes [2]. Ciprofloxacin is one of the three antibiotics recommended for chemoprophylaxis worldwide [3]. A number of ciprofloxacin-resistant meningococcal isolates have emerged in some countries since 1992 [4–8], although they remain rare in other countries. Resistance is mostly due to point mutations in the quinolone-resistance-determining region (QRDR) of the *gyrA* gene, which codes for subunit A of DNA gyrase [9], with a minor proportion attributed to efflux pump mechanisms [10,11].

In China, meningococcal disease is notifiable, with specimens and isolates sent to the local Center for Disease Control and Prevention (CDC). Since 1950, the Shanghai CDC has acquired data on the incidence and case fatality rates for meningococcal disease and has collected bacterial isolates from patients and their close contacts when available. Meningococcal disease was highly prevalent in China before the 1990s [12], and Shanghai was one of the high incidence zones in the country, with a peak incidence of 434 cases per 100,000 population in 1967 [13]. From 2003 to 2005, an unusual increase of serogroup C meningococcal cases was reported in Anhui Province, China, including 264 cases with 18 deaths. The hypervirulent lineage ST-4821 complex (CC4821) was first identified during this period and appeared to be responsible for ten of the serogroup C outbreaks, accounting for 72 cases [14]. In response, serogroup A and C polysaccharide vaccine has been used in China for routine immunization since 2006 [15].

Fluoroquinolones, including ciprofloxacin and norfloxacin, have been recommended for meningococcal chemoprophylaxis for adult close contacts of cases in China since 2005, with an oral dose of 100 mg every 8 h for 2 d. However, fluoroquinolones have been widely used for the treatment of other infectious conditions including infectious diarrhea, urinary tract infection, and respiratory tract infection since 1986 [16–21]. Between 2001 and 2010, fluoroquinolone consumption increased by approximately 2 × 10^8^ standard units and has become the third most consumed antibiotic in China [22]. Ciprofloxacin resistance was found to be highly prevalent (71%, 12/17) in serogroup W *N*. *meningitidis* isolates in Anhui Province during 2012–2013 [23]. However, data are scarce regarding the changes in serogroups, CCs, and antimicrobial susceptibility in *N*. *meningitidis* between the periods before and after the introduction of fluoroquinolones (the pre-quinolone and quinolone eras).

The goal of this study was to investigate the shifts in antimicrobial susceptibility, serogroups, and CCs of *N*. *meningitidis* associated with the introduction and expanding use of fluoroquinolones in Shanghai, using isolates from cerebrospinal fluid (CSF) and/or blood of invasive meningococcal disease cases that occurred in Shanghai in the pre-quinolone and quinolone eras and that were archived by the Shanghai CDC. Carriage isolates from pharyngeal carriage surveys conducted by the Shanghai CDC in both eras were also included to assess circulation of ciprofloxacin-nonsusceptible *N*. *meningitidis* in the healthy population.

## Methods

### Ethical Aspects

All specimens from meningococcal cases and close contacts were collected as part of the routine clinical management of patients, according to the national guidelines in China. Consequently, informed consent was not sought, and the study was approved by the Institutional Review Board of Huashan Hospital, Fudan University.

### Study Settings

The study was conducted in Shanghai, a municipality with province-level status and also the economic and financial center of China. It lies on China’s central eastern coast at the mouth of the Yangtze River, with over 20 million permanent residents residing in an area of 6,340 km^2^. With four distinct seasons, Shanghai is characterized as having a subtropical monsoon climate with an annual average temperature of 16°C. Since 1950, the Shanghai CDC has continuously acquired data on the incidence of and case fatality rates from meningococcal disease. *N*. *meningitidis* isolates during the periods of 1965–1985 and 2005–2013 were collected, with an interruption during 1986–2004. Because fluoroquinolones were introduced into clinical practice in 1986 locally [22,24], we characterized the meningococcal disease isolates from 1965–1985 and 2005–2013, corresponding to the pre-quinolone and quinolone eras, respectively.

### Meningococcal Surveillance in Shanghai

According to the National Notifiable Disease Surveillance System in China, a patient with a sudden onset of headache, fever, nausea, vomiting, petechial rash, or stiff neck, accompanied by delirium, coma or shock, or Gram-negative diplococci identified in the CSF defines a suspected case of meningococcal disease, which should be reported to the local CDC within 6 h. Specimens of all kinds (mainly CSF and blood) are cultured, and PCR is used for meningococcal identification if the cultures are negative. Under this mandate, all of the specimens and *N*. *meningitidis* isolate cultures from the CSF and/or blood of suspected cases in Shanghai have been sent to the Shanghai CDC since 2006. Before 2006, suspected cases of meningococcal disease were reported to the local CDC, but it was not mandatory to send the specimens or isolates to the CDC. Therefore, the Shanghai CDC collected and saved isolates only when the treating physicians elected to submit the specimens or isolates. During 1985–2004, a decrease in the incidence of meningococcal disease and the increased ability of hospital laboratories to culture and serogroup *N*. *meningitidis* without assistance from the Shanghai CDC resulted in interrupted collection of meningococcal isolates during this period.

For the present study, a confirmed case of meningococcal disease was defined by isolation of *N*. *meningitidis* from sterile sites (mainly CSF or blood), including culture-negative cases positive by Gram stain, latex agglutination, or PCR [14]. Close contacts were defined as those with history of working, living, studying, or nursing with a confirmed case, mainly including the household members, classmates, and roommates of the patients. Asymptomatic carriers were defined as healthy individuals with a pharyngeal swab growing *N*. *meningitidis* but without an epidemiological link to meningococcal cases.

### Carriage Surveys

During 1966–1978, surveys of meningococcal carriage were performed by the Shanghai CDC annually except in 1968 and 1969 (11 surveys total). Each pharyngeal swab was taken by swabbing the posterior pharyngeal wall behind the uvula through the mouth (posterior pharyngeal swab), using a dry cotton-tipped wooden swab held with a copper rod. Samples were plated directly onto chocolate agar plates supplemented with vancomycin (3.3 μg/ml) and polymyxin B (25 IU/ml), and delivered to the laboratory within 4 h. A total of 23,083 swabs were collected, from toddlers in child care centers, students in schools, and people working in crowded circumstances, such as sales associates in department stores, railway station workers, and uniformed service members in the army. Only some isolates from these surveys were kept in storage and were thus available for this study.

Additionally, surveys of pharyngeal carriage were conducted in September 2007 and September 2010, prior to the epidemic seasons of meningococcal disease (winter and spring). Both of the surveys were performed in three districts of Shanghai to cover 630 people from seven age groups (<1 y, 1–2 y, 3–4 y, 5–6 y, 7–14 y, 15–19 y, and ≥20 y), with 30 individuals sampled per age group per district. The age groups <1 y and 1–2 y comprised infants living at home, who were swabbed when they presented to local community health centers for scheduled immunization. The age groups 3–4 y and 5–6 y were preschool children, mostly in kindergarten. The age groups 7–14 y and 15–19 y were students in different grades, while ≥20 y comprised teachers working in primary school. Each posterior pharyngeal swab was taken with a CLASSIQSwab (Copan), an absorbent cotton-tipped plastic swab, and was processed as described above.

### Meningococcal Isolates and Bacterial Identification

The 374 isolates in this study comprised 305 isolates collected in 1965–1985 and 69 collected in 2005–2013. In 1965–1985, there were 126 disease isolates, 15 isolates from close contacts, and 164 carriage isolates. In 2005–2013, there were 37 disease isolates, 17 isolates from close contacts, and 15 carriage isolates. All of the isolates were confirmed as *N*. *meningitidis* by *Neisseria*-*Haemophilus* (NH) cards of the Vitek 2 automated system (bioMérieux). Serogroups were determined by slide agglutination using monoclonal antiserum to capsular polysaccharides A, B, C, W, X, Y, and Z (BD). Since 2007, PCR targeting *ctrA* and *porA* genes has been used for meningococcal identification of culture-negative cases [25,26]. PCR with primers for all 12 serogroups was used for serogrouping of culture-negative specimens and non-groupable strains [25].

### Antimicrobial Susceptibility Testing

The minimum inhibitory concentrations (MICs) of 11 antimicrobial agents were determined by the agar dilution method, with susceptibility interpreted according to the 2013 guidelines of the Clinical and Laboratory Standards Institute [27]. Ciprofloxacin-nonsusceptible strains (MIC ≥ 0.12 μg/ml as resistant and MIC = 0.06 μg/ml as intermediate) were investigated for efflux pump activity by determination of ciprofloxacin MIC with the microdilution method in the presence and absence of 20 μg/ml reserpine (Sigma-Aldrich) [10]. Decreased susceptibility to penicillin (MIC ≥ 0.12 μg/ml) was confirmed by Etest (bioMérieux).

### Molecular Typing for *N*. *meningitidis*

All isolates were characterized by MLST, typing of the variable regions of porin A (*porA* subtyping), and pulsed-field gel electrophoresis (PFGE) with the restriction endonuclease NheI (TaKaRa), as previously described [14,28]. The sequences of seven housekeeping genes (*abcZ*, *adk*, *aroE*, *fumC*, *gdh*, *pdhC* and *pgm*) were submitted to the *Neisseria* PubMLST database for assignment of sequence types (STs) [29]. A CC was defined as a group of related isolates whereby six or seven alleles are shared with at least one other member of the group [30]. In the same CC, single-locus variants (SLVs) represent two STs differing by one locus, and double-locus variants (DLVs) represent two STs differing by two loci. Clustering of PFGE patterns was assigned using the BioNumerics software package (version 6.0; Applied Maths), using the unweighted pair group method and an arithmetic averages (UPGMA) clustering algorithm [31], which was also used to construct dendrograms and minimum spanning trees based on MLST. To facilitate comparison of strains with different PFGE patterns, we used the method recommended by Popovic et al. for the investigation of meningococcal clonal dissemination [31]. With the position tolerance set at 1.5%, strains that had >95%, 85%–95%, and <85% relatedness were assigned to the same PFGE subtype, the same PFGE type, and different PFGE types, respectively, which represented strains that were closely related, possibly related, and genetically unrelated, respectively [31].

### Sequencing of Quinolone-Resistance-Associated Genes

Previously described primers and procedures were used to amplify and sequence the QRDRs of the gyrase genes (*gyrA* and *gyrB*) and the topoisomerase IV genes (*parC and parE*) [3,9]. The *mtr*R gene, a negative transcriptional regulator of the MtrCDE efflux pump, was also sequenced. If any amino acid substitutions or insertions/deletions were found in MtrR, efflux pump involvement was considered [32]. In order to study the polymorphism of the *gyrA* QRDR, *gyrA* (115–639) genes differing by at least one nucleotide were assigned a unique *gyrA* allele [9]. The Lasergene software package (version 7.1; DNASTAR), Clustal X, and MEGA 5 were used to analyze nucleotide sequences and the deduced amino acid sequences [3]. The PFGE data of the 2004 Anhui outbreak isolate 053442 (GenBank accession number CP000381) was provided by PulseNet China [33,34].

### Statistical Analyses

The incidence of meningococcal disease was calculated for 1950 to 2013 using population estimates from the Department of Acute Infectious Diseases of the Shanghai CDC. The chi-squared test for trend was used to determine linear trends of incidence over time, while regression splines were used to model nonlinear trends of incidence and case fatality rates during the period. Regression splines were performed using R (version 3.1.2; http://cran.r-project.org/). Incidences, case fatality rates, gender, age, predominant serogroups, predominant CCs, and susceptibility to ciprofloxacin, trimethoprim-sulfamethoxazole, and penicillin were compared between 1965–1985 and 2005–2013 for patients with meningococcal disease, close contacts, and asymptomatic *N*. *meningitidis* carriers using the chi-squared test (Fisher’s exact or Pearson’s). The odds ratio and 95% CI were calculated using the software OpenEpi module PersonTime2 (http://www.openepi.com/PersonTime2/PersonTime2.htm) [35]. Statistical analysis was performed using SPSS (version 20.0; IBM), and statistical significance was assessed at *p* < 0.05.

## Results

### Incidence and Case Fatality Rates of Meningococcal Disease in Shanghai

**Fig 1:**
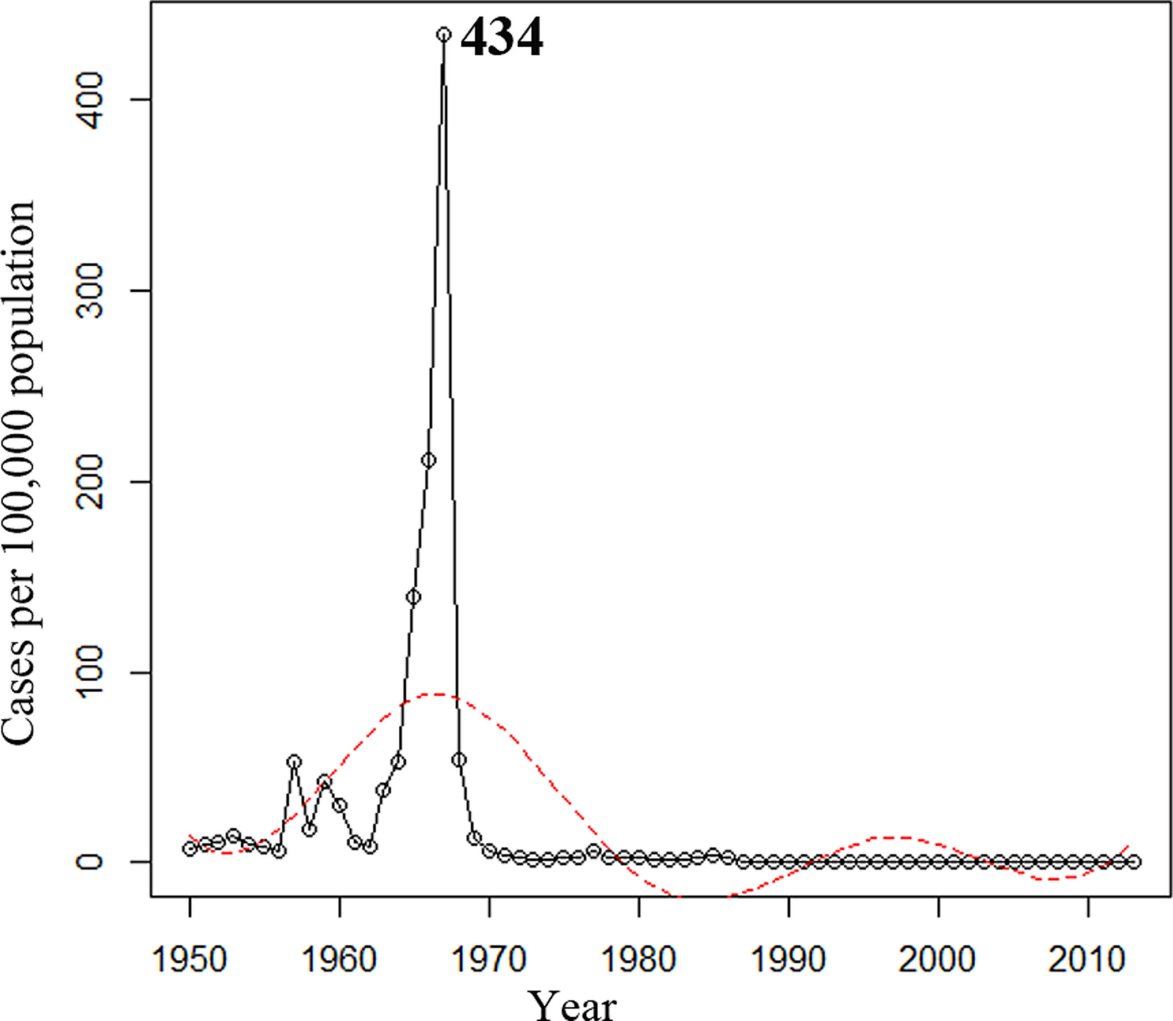
Annual incidence of reported cases of meningococcal disease in Shanghai from 1950 to 2013. The trend was analyzed using regression splines (red dotted line).

During the 64-y period from 1950 to 2013, 127,926 *N*. *meningitidis* cases were reported in Shanghai: 16,249 in 1950–1960, 105,671 in 1961–1970, 3,411 in 1971–1980, 1,957 in 1981–1990, 378 in 1991–2000, and 260 in 2001–2013, with the average incidences of 21.4, 97.6, 3.1, 1.6, 0.2, and 0.1 cases per 100,000 population for each period, respectively. The annual incidence peaked at 434 cases per 100,000 population in 1967 (Fig 1). The annual incidence for 1950–2013 was analyzed by regression spline (Table 1). The annual incidence fluctuated with time (Fig 1), with two waves observed in the 1950s–1980s and the 1980s–2000s. During 1965–1985, the average incidence was 43 cases per 100,000 population, compared with 0.08 during 2005–2013 (*p* < 0.001; Table 2).

**Table 1:** Regression splines of annual incidence and case fatality rate of meningococcal disease for 1950–2013 in Shanghai.

Parameter	Source	Standard Deviation Squared	Degrees of Freedom	Mean Squared	F-Statistic	*p*-Value
**Annual incidence**	Regression	1,182.84	6	197.14	3.54	0.005
	Residual error	3,174.33	57	55.69	NA	NA
	Total	4,357.17	NA	NA	NA	NA
**Annual case fatality rate**	Regression	2,415.48	6	402.58	65.46	<0.001
	Residual error	350.55	57	6.15	NA	NA
	Total	2,766.03	NA	NA	NA	NA

NA, not applicable.

**Table 2:** Epidemiological information and antimicrobial susceptibility of meningococcal isolates from 1965–1985 and 2005–2013.

Characteristic	Patients			Contacts and Carriers^a^		
	1965–1985 (*n* = 126)	2005–2013 (*n* = 37)	*p*-Value^b^	1965–1985 (*n* = 179)	2005–2013 (*n* = 32)	*p*-Value^b^
Incidence of reported meningococcal cases (per 100,000 population)	43, range 1–434	0.08, range 0.01–0.2	<0.001	ND	ND	ND
Case fatality rate (percent)	3, range 2.7–9.3	15, range 0–16.7	<0.001	ND	ND	ND
Male	65 (52)	23 (62)	0.17	125 (70)	22 (69)	ND
Age < 16 y	45 (36)	26 (70)	<0.001	10 (6)	12 (38)	0.53
Predominant serogroup	A (92 [73])	C (18 [49]); B (12 [32])	<0.001	B (93 [52])	B (22 [69])	<0.001
Predominant CC	CC5 (50 [40]); CC1 (41 [33])	CC4821 (23 [62])	<0.001	None	CC4821 (11 [34])	0.06
Ciprofloxacin-nonsusceptible strains^c^	0	31 (84)	<0.001	0	20 (63)	0.01
Trimethoprim-sulfamethoxazole-resistant strains^d^	117 (93)	35 (95)	0.54	149 (83)	30 (94)	<0.001
Penicillin-intermediate strains^e^	0	1 (3)	0.23	13 (7)	3 (9)	0.108

Data are *n* (percent) unless otherwise indicated.

^a^The numbers of close contacts and asymptomatic carriers were 32 and 179, respectively.

^b^Chi-squared test was used (Fisher’s exact or Pearson’s) to analyze the disparity of isolates between 1965–1985 and 2005–2013. *p*-Value < 0.05 indicates statistical significance.

^c^The ciprofloxacin MIC range, MIC_50_, and MIC_90_ were 0.015–0.25, 0.015, and 0.125 μg/ml, respectively.

^d^The trimethoprim-sulfamethoxazole MIC range, MIC_50_, and MIC_90_ were 0.015/0.3–4/76, 1/19, and 2/38 μg/ml, respectively.

^e^The penicillin MIC range, MIC_50_, and MIC_90_ were 0.015–0.25, 0.03, and 0.06 μg/ml, respectively.

ND, not determined.

**Fig 2:**
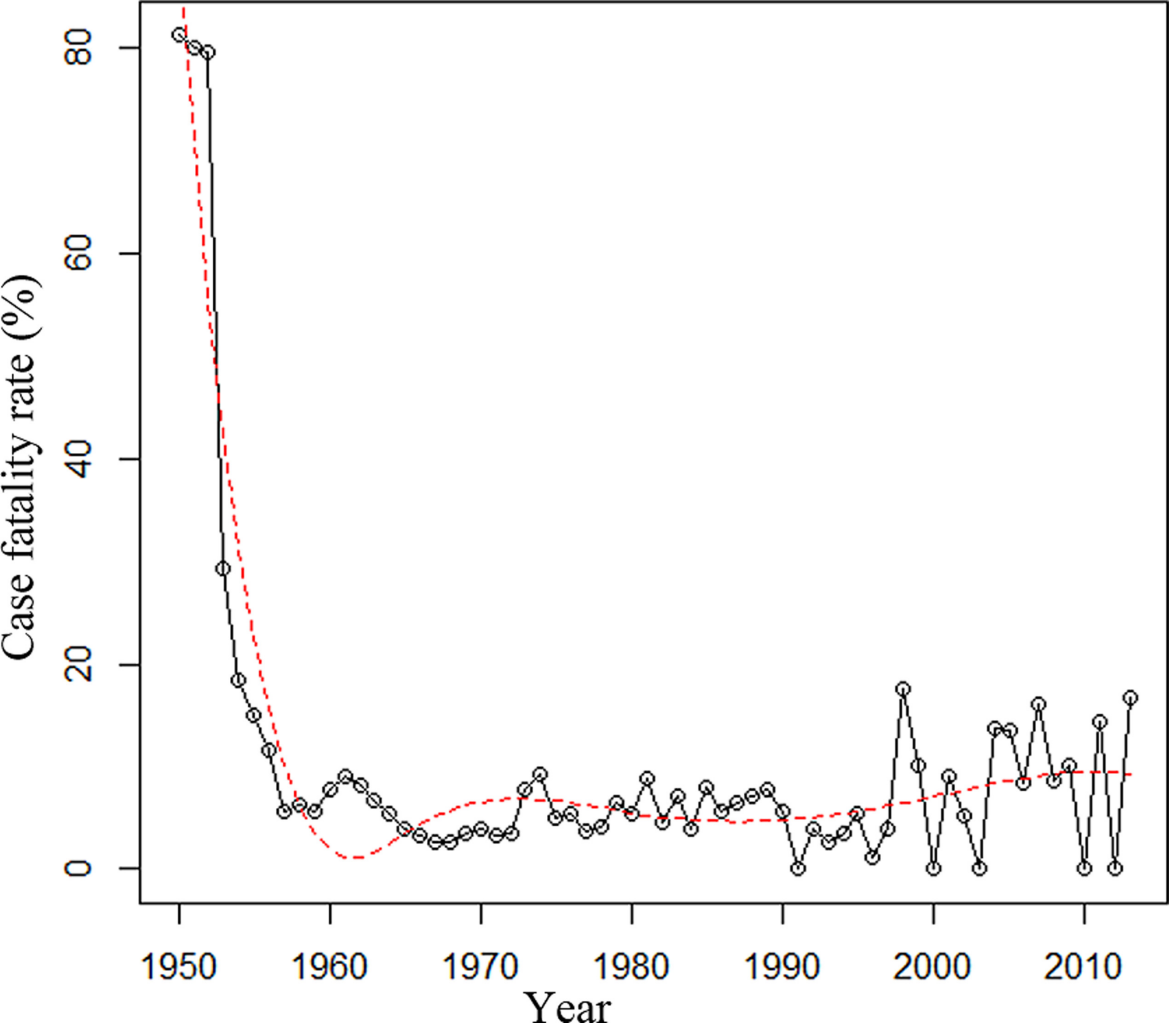
Case fatality rate of reported cases of meningococcal disease in Shanghai from 1950 to 2013. The trend was analyzed using regression splines (red dotted line).

The case fatality rate of meningococcal disease was 15% in 1950–1960, 3% in 1961–1970, 5% in 1971–1980, 7% in 1981–1990, 4% in 1991–2000, and 10% in 2001–2013. The trend of case fatality rate over time for 1950–2013 was analyzed (Table 1). The case fatality rate was relatively stable after a sharp decrease during the 1950s (Fig 2).

### Clonal Complexes of *N*. *meningitidis* Disease Isolates over Time

**Fig 3:**
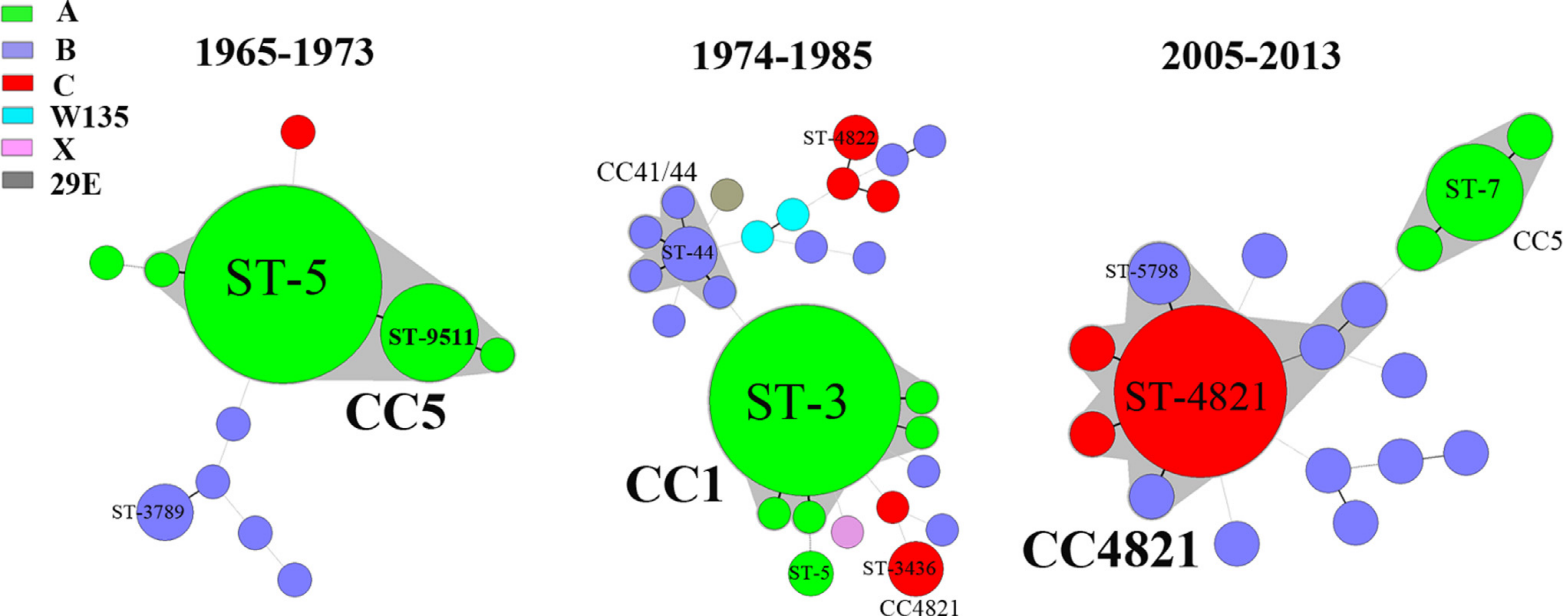
Minimum spanning tree analysis of multi-locus sequence types of *N*. *meningitidis* from patients. Isolates were obtained from patients during the periods 1965–1973, 1974–1985, and 2005–2013. STs are displayed as circles. The size of each circle indicates the number of isolates of this particular type. Serogroup is distinguished by different colors. The shaded halo surrounding the STs encompasses related STs that belong to the same CC. Heavy solid lines represent SLVs, and light solid lines represent DLVs.

Of the 126 *N*. *meningitidis* disease isolates from 1965–1985 that were available for this study, serogroup A was predominant (73%, 92/126), followed by B (17%) and C (7%). The serogroup A ST-5 complex/subgroup III complex (CC5; 84%, 48/57) was predominant in 1965–1973, and was responsible for the epidemic in 1967, while serogroup A ST-1 complex/subgroup I/II complex (CC1; 58%, 40/69) predominated in 1974–1985 (Fig 3).

During 2005–2013, among the 125 suspected cases of meningococcal disease reported in total, 58 cases were confirmed, 47 by culture and 11 by PCR. With the exception of ten isolates from 2005 that became nonviable during storage in the hospital laboratory, 37 disease isolates were available for characterization. Serogroup C (49%, 18/37) was predominant, followed by serogroup B (32%, 12/37) and A (19%, 7/37). The ST-4821 complex (CC4821; 62%, 23/37) of serogroup C (*n* = 18) or B (*n* = 5) was predominant (Fig 3), while all seven serogroup A strains were assigned to CC5.

### Carriage Surveys

During 1965–1985, 11 surveys of meningococcal carriage were performed. A total of 2,402 meningococcal isolates were isolated from the 23,083 pharyngeal swabs, with an average carriage rate of 10% (range, 2% in 1972 to 24% in 1967). The proportion of serogroup A strains in 1966 and 1967 was more than 70%; however, thereafter it decreased from 50% in 1970 to 0.5% in 1978 (*p* < 0.001), accompanied by an increase in serogroup B, serogroup C, or other serogroups (S2 Table). Only 164 isolates from these surveys were kept in storage and were thus available for this study.

Two surveys were performed during 2005–2013. In 2007, a total of 553 pharyngeal swabs were collected from healthy individuals with a median age of 5 y (range, 3 mo to 49 y). *N*. *meningitidis* was isolated from 11 survey participants (2%), from two age groups: 7–14 y (10%, 9/90) and ≥20 y (2%, 2/94). In 2010, 644 individuals with a median age of 5 y (range, 3 mo to 54 y) were investigated. Four (0.6%) were positive for *N*. *meningitidis* carriage, all in the age group ≥20 y (4%, 4/92). Among the 15 isolates from the two carriage surveys, 13 (87%) were serogroup B, one was W, and one was non-groupable, while no serogroup A or C strains were found (S2 Table).

### Antimicrobial Susceptibility

A total of 374 *N*. *meningitidis* isolates were tested for antimicrobial susceptibility, including the 163 disease isolates, 32 isolates from close contacts, and 179 asymptomatic carriage isolates. All meningococcal isolates from meningococcal cases in 1965–1985 (*n* = 126) were susceptible to ciprofloxacin (MIC ≤ 0.015 μg/ml for all 126 isolates), while 84% (31/37) of the disease isolates from 2005–2013 (*p* < 0.001) were ciprofloxacin-nonsusceptible (Table 2), including 30 resistant and one intermediate isolates (MIC = 0.06 μg/ml [*n* = 1], 0.125 μg/ml [*n* = 29], and 0.25 μg/ml [*n* = 1]; MIC_50_ and MIC_90_ were both 0.125 μg/ml). The 31 ciprofloxacin-nonsusceptible strains had nalidixic acid MICs ranging from 16 to 128 μg/ml. The resistance frequencies for trimethoprim-sulfamethoxazole were 93% and 95% in 1965–1985 and 2005–2013 (*p* = 0.13), respectively. Only one isolate in 2005–2013 was intermediately resistant to penicillin (MIC = 0.38 μg/ml).

During 1965–1985, all of the 179 isolates from 15 close contacts and 164 asymptomatic carriers were ciprofloxacin susceptible (MIC ≤ 0.015 μg/ml), while 76% (13/17) of close contact isolates and 47% (7/15; five in 2007 and two in 2010) of carriage isolates (*p* = 0.005) in 2005–2013 were ciprofloxacin-nonsusceptible (MIC = 0.06–0.25 μg/ml). The frequency of resistance to trimethoprim-sulfamethoxazole was 83% in 1965–1985 and 94% in 2005–2013 (*p* = 0.10). Among the isolates from close contacts, the penicillin intermediate (MIC = 0.125–0.25 μg/ml) rate was 0% and 6% (1/17) during the two periods (*p* = 0.52), respectively, while among the carriage isolates, the rate was 8% (13/164) and 13% (2/15) during the two periods (*p* = 0.45), respectively (Table 2).

All 374 isolates were susceptible to the other antimicrobial agents tested, including cefotaxime, ceftriaxone, meropenem, chloramphenicol, azithromycin, minocycline, and rifampin.

### Characterization of 31 Ciprofloxacin-Nonsusceptible Meningococcal Cases

Thirty-one cases of invasive meningococcal disease from February 2005 to April 2013 for which the isolates were available were caused by ciprofloxacin-nonsusceptible strains, including 17 serogroup C, seven serogroup B, and seven serogroup A. Patients were distributed across 12 of the 17 districts of Shanghai, especially in the central urban area (S1 Fig), and two cases in 2013 were likely imported cases from Jiangsu Province and Hong Kong. The median age of the patients was 4 y (range, 2 mo to 43 y), 13 (42%) were students, 20 (65%) were male, and all denied recent foreign travel. Fourteen patients had been immunized with serogroup A polysaccharide vaccine; ten of them were infected with serogroup C strains, one with a serogroup A strain, and three with serogroup B strains. Seven patients had not received any meningococcal vaccine, and there was no immunization record available for ten patients. The investigation identified no common exposures, social settings, or other epidemiological relationship among these patients.

Based on the classification system of clinical presentation [36], the clinical symptoms of 31 ciprofloxacin-nonsusceptible meningococcal disease cases could be divided into three groups, namely, fulminant meningococcal septicemia (48%, 15/31), distinct meningitis (32%, 10/31), and distinct meningitis and persistent septic shock (19%, 6/31). Despite appropriate treatment with penicillin, ceftriaxone, or meropenem, three (10%) patients died.

**Fig 4:**
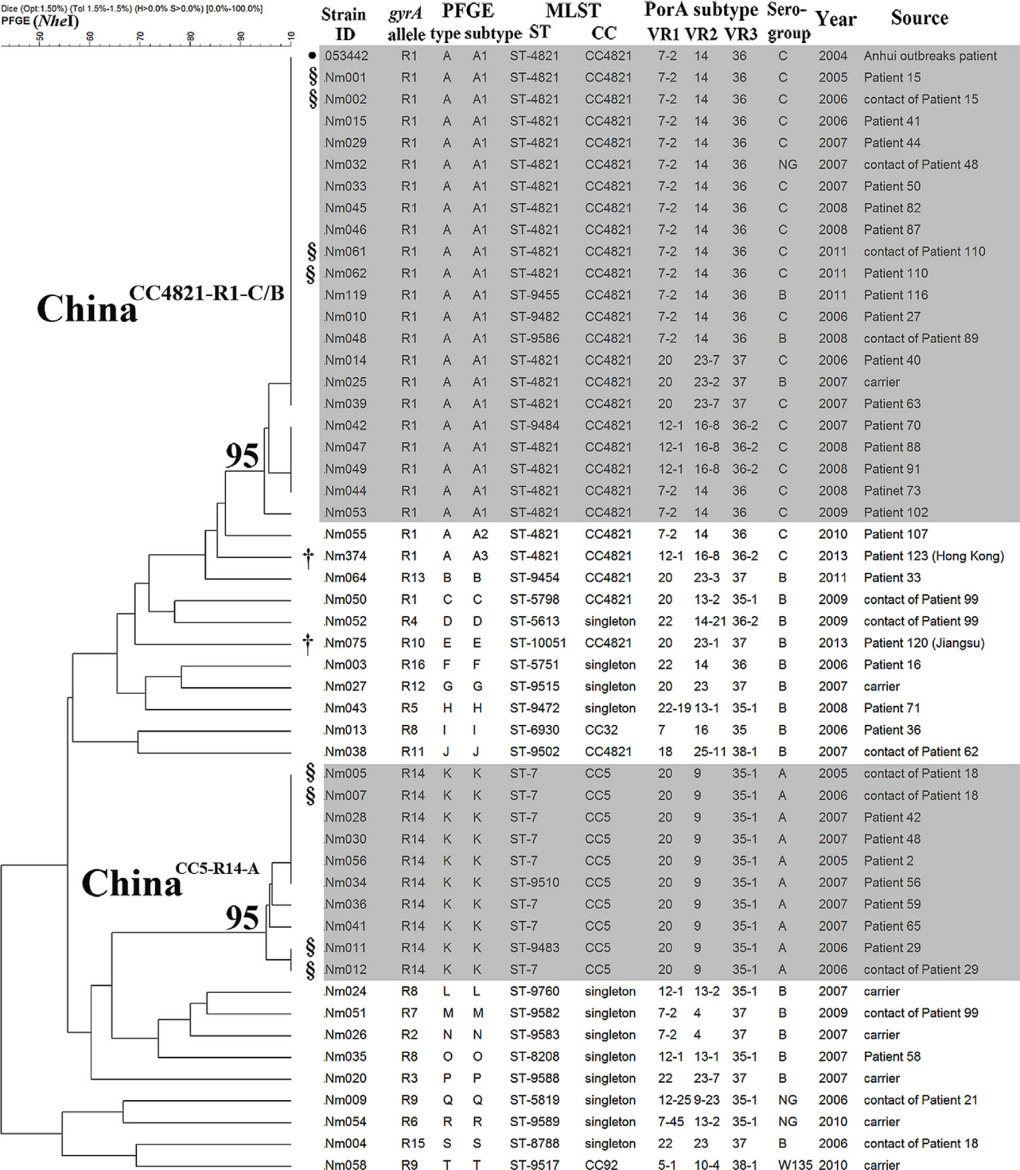
Dendrogram of 51 ciprofloxacin-nonsusceptible *N*. *meningitidis* strains from patients and carriers in Shanghai, China, 2005–2013, constructed using pulsed-field gel electrophoresis—NheI. The clones China^CC4821-R1-C/B^ and China^CC5-R14-A^ are indicated in grey shading, with the relatedness values marked on the roots. ●The PFGE data for the Anhui outbreak strain 053442 were obtained from PulseNet China. §Clonal dissemination between patients and their close contacts was observed. †Imported cases. NG, non-groupable.

Close contacts of all 31 patients were investigated, and no additional meningococcal infections were detected. Among the 17 close contacts with carriage of *N*. *meningitidis* after 2005, one was administered norfloxacin, and the other 16, including two children, were given oral cefradine (*n* = 9) or trimethoprim-sulfamethoxazole (*n* = 7). Thirteen close contacts (age range 8 to 59 y) of nine cases, including five cases without proof of meningococcus by culture or PCR (clinically diagnosed meningococcal cases), were found to carry ciprofloxacin-nonsusceptible strains by pharyngeal swab culture (Fig 4).

### *gyrA* Mutations in 51 Ciprofloxacin-Nonsusceptible Strains

Mutations in the QRDR of *gyrA* associated with quinolone resistance substitutions were observed in all of the 51 ciprofloxacin-nonsusceptible strains from patients (*n* = 31), close contacts (*n* = 13), and asymptomatic carriers (*n* = 7), of which 49 harbored the typical substitution of threonine to isoleucine at amino acid position 91 (T91I). The other two ciprofloxacin-intermediate strains (MIC = 0.06 μg/ml) harbored aspartate to asparagine substitutions at amino acid position 95 (D95N) (Table 3). No additional mutations were observed in the QRDRs of *gyrB*, *parC*, or *parE*. In addition, there were no ciprofloxacin-nonsusceptible isolates with more than a 4-fold reduction in MIC for ciprofloxacin in the presence of reserpine, or with missense or nonsense mutations in *mtrR*.

**Table 3:** *gyrA* alleles in 51 ciprofloxacin-nonsusceptible *N*. *meningitidis* strains.

*gyrA* Allele^a^	Number of Strains	*gyrA* Allele in PubMLST	MLST Profile^b^	MIC (μg/ml)	Amino Acid Alteration(s)	Nucleotide Change of T91I	GenBank Accession Number
R1	24	71	CC4821	0.06–0.125	T91I	ACC→ATC	KJ415179–KJ415202
R2	1	10	ST-9583	0.06	D95N	No	KJ415203
R3	1	New	ST-9588	0.06	T91I	ACC→ATC	KJ415204
R4	1	New	ST-5613	0.25	T91I	ACC→ATC	KJ415228
R5^c^	1	New	ST-9472	0.125	T91I	ACC→ATC	KJ415205
R6^c^	1	New	ST-9589	0.06	D95N	No	KJ415206
R7	1	New	ST-9582	0.125	T91I	ACC→ATC	KJ415207
R8	3	New	CC32, ST-9760, ST-8208	0.125	T91I	ACC→ATC	KJ415208
R9	2	New	CC92, ST-5819	0.125	T91I	ACC→ATC	KJ415209
R10	1	New	CC4821	0.125	T91I, N103D, I111V	ACC→ATC	KJ415210
R11^c^	1	New	CC4821	0.25	T91I, N103D, I111V	ACC→ATC	KJ415211
R12	1	New	ST-9515	0.125	T91I, N103D, I111V	ACC→ATT	KJ415212
R13	1	New	CC4821	0.125	T91I, N103D, I111V	ACC→ATT	KJ415213
R14	10	13	CC5	0.125	T91I, N103D, I111V	ACC→ATT	KJ415214–KJ415223
R15	1	New	ST-8788	0.125	T91I, N103D, A105S	ACC→ATA	KJ415229
R16	1	New	ST-5751	0.125	T91I, N103D, A105S	ACC→ATA	KJ415230

^a^ *gyrA* alleles were defined in this study based on nucleotides 115–639 of the *gyrA* gene.

^b^Isolates assigned to any CC are shown with CC, while the isolates unable to be assigned are shown with ST.

^c^Additional alterations in ParE: R5, H495N; R6, A450V; R11, A443T and D480N.

New, unable to assign in the PubMLST database.

Sixteen *gyrA* alleles (R1–R16) were defined in 51 ciprofloxacin-nonsusceptible isolates (Table 3), and 12 alleles (S1–S12) in 323 ciprofloxacin-susceptible isolates (GenBank accession numbers KJ415179 to KJ415238). A phylogenetic analysis was performed using the *gyrA* QRDRs from 658 *N*. *meningitidis*, *N*. *gonorrhoeae*, and *N*. *lactamica* isolates, which included the 374 isolates in this study and 284 isolates from GenBank and the *Neisseria* PubMLST database. Isolates with *gyrA* alleles S1–S12 and R1–R2 were grouped within the *N*. *meningitidis* cluster, R3–R9 within the *N*. *lactamica* cluster, R10–R16 outside of the three *Neisseria* clusters, and none were grouped within the *N*. *gonorrhoeae* cluster (S2 Fig).

### Identification of Two Predominant Ciprofloxacin-Nonsusceptible Clones

Among the 31 ciprofloxacin-nonsusceptible isolates from patients, 20 (65%) were assigned to CC4821, seven (23%) to CC5, and four to either singletons (*n* = 3) or CC32 (*n* = 1). Among the 13 ciprofloxacin-nonsusceptible strains from close contacts, six were assigned to CC4821 and three to CC5, while the remaining four isolates were singletons. Among the seven ciprofloxacin-nonsusceptible strains from asymptomatic carriers, five isolates were assigned to five different singletons and two isolates to two different CCs (CC4821 and CC92) (Fig 4).

Of 34 CC4821 strains in 2005–2013, 27 (79%) were ciprofloxacin-nonsusceptible with substitution T91I in GyrA, whereas all 18 CC4821 strains from 1965–1985 were ciprofloxacin-susceptible (*p* < 0.001). There were 21 ciprofloxacin-nonsusceptible CC4821 strains with PFGE patterns closely related (relatedness > 95%) to the Anhui outbreak strain 053442, representing a major outbreak clone (Fig 4). We named the clone China^CC4821-R1-C/B^, characterized as having MLST profile ST-4821 or its SLV, plus *gyrA* allele R1, serogroup C or B (except one non-groupable), and PFGE subtype A1 (Fig 4 and S3 Fig). All the *gyrA* R1 alleles of the 21 China^CC4821-R1-C/B^ strains were grouped within the *N*. *meningitidis* cluster in the phylogenetic analysis. In the complete-length *gyrA* gene (2,751 nucleotides), only one nucleotide change (leading to T91I) was found among the China^CC4821-R1-C/B^ strains from the ciprofloxacin-susceptible CC4821 strain with the S1 allele isolated early in 1972.

All ten CC5 strains in 2005–2013 were ciprofloxacin-resistant, while all 63 CC5 strains isolated during 1965–1985 were ciprofloxacin-susceptible (*p* < 0.001). The ten ciprofloxacin-resistant CC5 strains showed a PFGE relatedness of more than 95%, representing another clone that we named China^CC5-R14-A^. This clone is characterized as having ST-7 or its SLV, *gyrA* allele R14, serogroup A, PorA subtype P1.20,9,35–1, and PFGE subtype K (Fig 4 and S3 Fig). In addition to the R14 allele, which grouped outside the *N*. *meningitidis* cluster, at least 141 nucleotides in the whole sequence of the *gyrA* gene were found to differ between resistant and susceptible CC5 strains.

### Circulation of Ciprofloxacin-Nonsusceptible Clones

Amongst those cases for which a viable isolate was available (37/47, 79%), ciprofloxacin resistance was evident in 31 cases of invasive meningococcal disease (with three deaths), among which 16 (52%) cases were caused by the China^CC4821-R1-C/B^ clone, seven (23%) by the China^CC5-R14-A^ clone, four by other CC4821 strains, one by CC32, and three by singleton strains. The cases were distributed in eight and five of the 17 districts in Shanghai, respectively (S1 Fig). No obvious epidemiological links were observed among these cases. However, transmission between patients and close contacts was presumed in at least four cases based on identification of the same clones in patients and the contacts (Fig 4).

The first case was a 21-y-old factory worker (Patient 15) hospitalized with CSF-culture-confirmed meningococcal meningitis during December 2005–January 2006. The second case was a 4-y-old child (Patient 110) with blood-culture-confirmed meningococcal septicemia in January 2011. Both patients were infected with the China^CC4821-R1-C/B^ clone and recovered with penicillin treatment. The same clone was also identified from the roommate of Patient 15 (Nm002) and the mother of Patient 110 (Nm061). The third case was a 4-mo-old infant (Patient 29) who was hospitalized with CSF-culture-confirmed meningococcal meningitis in April 2006 and recovered upon treatment with penicillin and ceftriaxone. An identical China^CC5-R14-A^ strain was isolated from a throat sample from his grandfather (Nm012). The fourth case was a 3-y-old boy (Patient 18), who was born in southwest China (Guizhou Province) then moved to Shanghai. He died of clinically diagnosed meningococcal meningitis in February 2006, without any specimens available for culture. Despite the absence of isolates from this patient to provide direct proof, the China^CC5-R14-A^ clone was recovered from both the patient’s father (Nm005) and uncle (Nm007), suggesting this clone was the culprit of the fatal disease in this child.

## Discussion

Here, we present shifts in *N*. *meningitidis* ciprofloxacin susceptibility, serogroup prevalence, and CC prevalence associated with the introduction and expanding use of quinolones in Shanghai, China, based on meningococcal isolates that had been stored and were available at the time of the study. The findings are distinct from studies on meningococcal quinolone resistance conducted in North America, Europe, South Africa, and other parts of China in several aspects [3,9,23,37,38]. Ciprofloxacin-nonsusceptible strains in Shanghai were found with much higher frequency in the quinolone era than in the pre-quinolone era, not only in patients but also in the healthy population, and the majority of isolates were associated with two epidemic Chinese clones, China^CC4821-R1-C/B^ and China^CC5-R14-A^, identified as circulating in Shanghai since 2005. Also, the strains acquired resistance by different means: via a point mutation in the *gyrA* gene and horizontal gene transfer from other *Neisseria* species (possessing different *gyrA* genes based on phylogenetic analysis).

Since the 1970s, the incidence of meningococcal disease in Shanghai has decreased rapidly. Since 1965, at least three clonal waves of *N*. *meningitidis* causing meningococcal disease in Shanghai have occurred. Serogroup A CC5 was epidemic in 1965–1973, while serogroup A CC1 was predominant in 1974–1985, and serogroup C or B CC4821 was predominant after 2005. These changes may have been promoted by meningococcal vaccination. From 1969 to 1980, children aged 1 to 6 y in Shanghai were immunized with serogroup A meningococcal whole cell vaccine. In 1980, national immunization with serogroup A polysaccharide vaccine was initiated in China, targeting children and adolescents aged 6 mo to 15 y with a three-dose schedule (at 6, 9, and 36 mo in infants) by subcutaneous injection in the upper arm [39]. At the same time, a catch-up campaign was implemented targeting high-risk populations, such as sales personnel in department stores, railway station workers, and soldiers in the army. Because of the Anhui serogroup C CC4821 outbreak during 2003–2005, the national meningococcal immunization program then expanded to include both serogroups A and C, with four injections in children aged 6 mo to 15 y. Polysaccharide vaccine A is administered to infants at age 6 and 9 mo, while polysaccharide vaccine A plus C is given at age 3 and 6 y.

The changes in meningococcal CCs in Shanghai reflect similar shifts taking place in the whole country. During the 1950s–1990s, the proportion of serogroup A was more than 95% in China [14], while in 2003–2008, it declined to 35%, and the proportion of serogroup C rose to 59% [40]. Correspondingly, CC4821 (55%) became predominant in China, followed by CC5 (37%) [40].

The carriage of serogroup A strains in the healthy population in Shanghai was found to have decreased significantly (*p* < 0.001) since 1970, just after the serogroup A vaccination campaign, and a low carriage (0.08%, 1/1,311) was also found in 2011 in Guangxi Province, China [15]. A similar trend has been observed in Africa, the main region of serogroup A meningococcal disease cases [1]. The incidence of serogroup A in South Africa decreased by 95% from 2003 to 2007 [41], and a similar decrease in the rate of carriage (94%) and incidence (95%) was observed in the African “meningitis belt” after the introduction of the serogroup A conjugate vaccine MenAfriVac in 2010 [42].

Furthermore, serogroup B and other serogroup strains, which comprised 30% (8/27) of the ciprofloxacin-nonsusceptible CC4821 strains (Fig 4), may have facilitated the spread of ciprofloxacin resistance, since no vaccines have been licensed in China to provide broad coverage that includes serogroup B meningococci. Bacterial dynamics should be monitored both in pharyngeal carriage of *N*. *meningitidis* and bacterial meningitis at the population level in order to prevent epidemics of these hypervirulent clones in the future.

This study found that up to 84% of *N*. *meningitidis* disease isolates were ciprofloxacin-nonsusceptible in 2005–2013 (quinolone era), even though quinolones were rarely used for meningococcal chemoprophylaxis in Shanghai during this study period. This nonsusceptibility rate was much higher than that observed in North Dakota and Minnesota in the United States between 2007 and 2008 (9%, 3/33) [3]. Besides in the disease isolates, a high frequency of ciprofloxacin nonsusceptibility was also found in isolates from asymptomatic carriers (47%) after 2005. In a report from the China CDC in 2014 [38], a ciprofloxacin nonsusceptibility rate of 72% (353/487) was mentioned, though no further details were provided. Current recommendations for meningococcal chemoprophylaxis in the national scheme for preventing and controlling meningococcal meningitis in China include the use of fluoroquinolones, cephalosporins, chloramphenicol, rifampin, or sulfafurazole, which have not been changed since 2005, when the scheme was approved by Ministry of Public Health of China. In practice, ciprofloxacin and amoxicillin were used as prophylaxis in the CC4821 outbreak (2004), and cefixime was used in a case of serogroup W (2012) in Anhui [43,44], while trimethoprim-sulfamethoxazole and cefradine tablets have been used in sporadic cases in Shanghai since 2006. Among these agents, fluoroquinolones should be used with caution for chemoprophylaxis based on the high frequency of ciprofloxacin-resistant *N*. *meningitidis* in China, given the presence of safe and effective alternatives such as ceftriaxone and rifampin. Sulfonamides and chloramphenicol should not be used, for the reason of either high frequency of resistance or frequent adverse effects. Azithromycin in a single 500-mg dose is effective to eradicate pharyngeal carriage and could be considered [4,45].

The high frequency of ciprofloxacin nonsusceptibility in Shanghai seems to be mainly linked with the emergence and spread of CC4821 and CC5 strains. Eighty-seven percent (27/31) of the ciprofloxacin-nonsusceptible strains from patients were assigned to either CC4821 or CC5, all with the substitution T91I in GyrA. In 2005–2013, CC4821 strains showed a ciprofloxacin nonsusceptibility rate of 79% (27/34), while all CC5 strains were resistant to ciprofloxacin. Given that CC4821 and CC5 strains not only accounted for 92% of meningococcal disease isolates in 2003–2008 in China [40] but also were responsible for a high proportion of ciprofloxacin-nonsusceptible cases, it is not surprising to find that ciprofloxacin-nonsusceptible *N*. *meningitidis* is highly prevalent in China [38]. Most of the CC4821 or CC5 strains are genetically homogenous, and we designated them China^CC4821-R1-C/B^ and China^CC5-R14-A^, respectively. We found that these specific clones have been circulating in Shanghai since 2005, disseminating among some patients and their close contacts and facilitating the spread of ciprofloxacin resistance.

**Fig 5:**
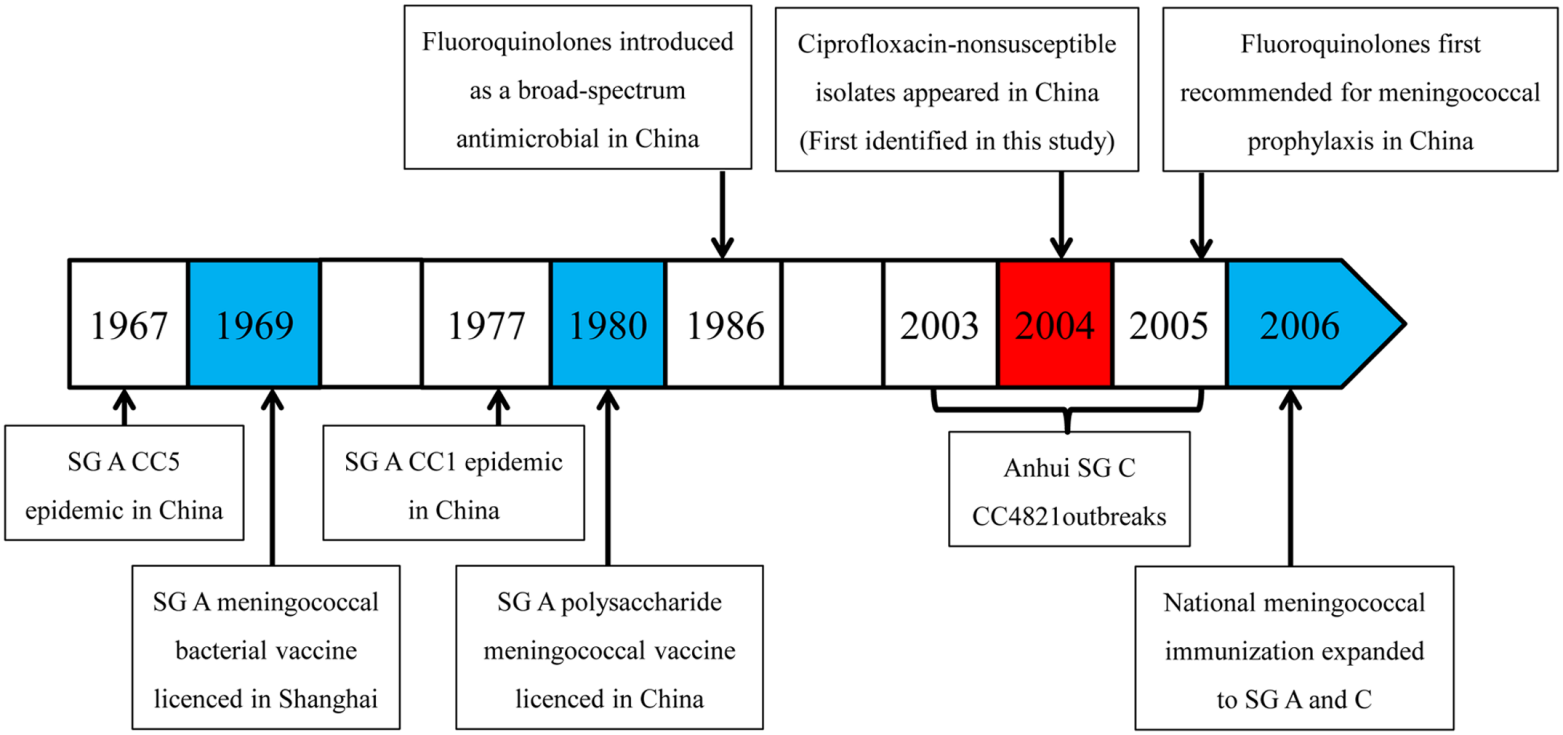
Timeline for the introduction of fluroquinolones and vaccines, and clonal waves of *N*. *meningitidis* in China. SG, serogroup.

The China^CC4821-R1-C/B^ clone is unique to China, and appears to have acquired ciprofloxacin resistance by a point mutation in *gyrA*, leading to an amino acid change of T91I in GyrA, compared with the whole sequence of *gyrA* in the early ST-4821 strain in 1972. The Anhui outbreak strain 053442 was the earliest China^CC4821-R1-C/B^ clone we could identify (Fig 5). It was isolated from the CSF sample of a patient in the Anhui outbreak in 2004, and possessed the typical molecular traits of the epidemic CC4821 strains [14,34]. By analyzing its *gyrA* sequence, we found that strain 053442 possessed the T91I in GyrA, with a ciprofloxacin MIC of 0.064 μg/ml (data provided by the China CDC), suggesting that fluoroquinolone resistance was already present during the Anhui outbreak.

The China^CC5-R14-A^ clone belongs to the hypervirulent lineage CC5, and might have acquired resistance to ciprofloxacin via uptake and incorporation of *gyrA* fragments with T91I from other meningococci or *Neisseria* species. Responsible for three pandemics of epidemic meningitis, the serogroup A CC5 strains have spread from China to many parts of the world since the 1960s [46]. It is worth noting that ciprofloxacin-resistant CC5 strains have also been reported in Europe (*n* = 7) and Israel (*n* = 1) between 2004 and 2011 (S1 Table). In Europe, three CC5 strains were identified in 2006 and 2009 [5,29] that are genetically closely related to the China^CC5-R14-A^ clone (ST-7 or its SLV, serogroup A, PorA subtype of P1.20,9, and *gyrA* allele R14).

Among the 51 ciprofloxacin-nonsusceptible *N*. *meningitidis* strains, 14 strains showed diverse MLST profiles, with 79% of them belonging to serogroup B. In 2014, 71% (12/17) of serogroup W isolates were reported to be ciprofloxacin-resistant in Anhui Province [23], including four isolates from two fatal cases and 13 from close contacts. All 17 of the isolates showed the same molecular characteristics of ST-11 and P1.5,2, which started to spread in southeastern China in 2011 [15]. In our study, a serogroup W ciprofloxacin-resistant strain of CC92 from a carrier in 2010 with the R9 *gyrA* allele grouped within the *N*. *lactamica* cluster.

A limitation of this study is that no isolates from the period 1986–2004 in Shanghai were available, and only a small number of isolates from 1965–1985 were available. However, a study performed by the Children’s Hospital of Fudan University in Shanghai provides insights for the period 1984–2003. In that study, a total of 67 isolates were isolated from patients (age range 23 d to 13 y). It was reported that among the 67 isolates studied, 50 were isolated during 1984–1993, including 26 (52%) serogroup A, 21 (42%) B, and three non-groupable strains, while the other 17 isolates were isolated during 1994–2003 and were not tested for serogroup. Twenty-three isolates were chosen randomly to determine antimicrobial susceptibility, and all were susceptible to fluoroquinolones [47]. Therefore, it is likely that ciprofloxacin resistance emerged after the Anhui outbreak during 2003–2005. It would be interesting in the future to compare the whole genomes of China^CC4821-R1-C/B^ strains with those of the early CC4821 strains to understand the pathogenesis and evolution in virulence underpinning the expansion of this hypervirulent clone.

Our findings demonstrate that between the pre-quinolone and quinolone eras, shifts in ciprofloxacin susceptibility, serogroups, and CCs have occurred in Shanghai, China. Since 2005, ciprofloxacin nonsusceptibility in *N*. *meningitidis* has become highly prevalent in Shanghai, in association with the spread of the hypervirulent lineages CC4821 and CC5. The dissemination of two ciprofloxacin-nonsusceptible clones, China^CC4821-R1-C/B^ and China^CC5-R14-A^, appears to have facilitated the spread of ciprofloxacin resistance.

## Supporting Information

S1 Checklist STROBE Statement. (DOC)

S1 Fig Geographic distribution of *N*. *meningitidis* with different ciprofloxacin susceptibilities in Shanghai from 2005 to 2013. Ciprofloxacin-nonsusceptible isolates are indicated in red, including 47 ciprofloxacin-resistant isolates and four ciprofloxacin-intermediate isolates. Ciprofloxacin-susceptible isolates are indicated in black. ▲, patient; ●, close contact; ○, asymptomatic carrier.
(TIF)

S2 Fig Phylogenetic analysis of *gyrA* quinolone-resistance-determining region from 658 *Neisseria* spp. isolates. Phylogenetic analysis of the QRDR of *gyrA* (nucleotides 115–518) from 586 *N*. *meningitidis* (NM), 52 *N*. *gonorrhoeae* (NG), 19 *N*. *lactamica* (NL), and one *N*. *cinerea* (NC) isolates from GenBank or the *Neisseria* PubMLST database was conducted in MEGA 5 using the unweighted pair group method with arithmetic mean averages (UPGMA). The percentages of replicate trees in which the associated taxa clustered together in the bootstrap test (2,000 replicates) are shown next to the main branches. The clusters were determined with the bootstrap values >70% [48]. Strains are shown as “strain number or *gyrA* allele/country or district/species/GyrA mutation or wild type/total number.” Strains with no alteration in GyrA were assumed to be wild type. The alleles labeled with wine-colored squares were defined among ciprofloxacin-nonsusceptible isolates from Shanghai, and those labeled with cyan circles were defined by Eva Hong et al. among ciprofloxacin-nonsusceptible *N*. *meningitidis* isolates from Europe [9]. Three species clusters are indicated.
(TIF)

S3 Fig Minimum spanning tree analysis of 374 *N*. *meningitidis* isolates from patients, close contacts, and asymptomatic carriers in Shanghai from 1965 to 2013. In the minimum spanning tree, the STs are displayed as circles. The size of each circle reflects the number of isolates within this particular type. The susceptibility to ciprofloxacin is represented by different colors. The colored halos surrounding the STs denote types that belong to the same CC. Heavy solid lines represent SLVs, light solid lines represent DLVs, heavy dotted lines represent triple-locus variants, and light dotted lines represent quadruple-locus variants.
(TIF)

S1 Table Published CC5 ciprofloxacin-resistant *N*. *meningitidis* strains outside China. (DOCX)

S2 Table Analysis of 13 surveys of meningococcal carriage in Shanghai from 1965 to 2013. (DOCX)

## Abbreviations

CC: clonal complex
CDC: Center for Disease Control and Prevention
CSF: cerebrospinal fluid
DLV: double-locus variant
MIC: minimum inhibitory concentration
MLST: multi-locus sequence typing
PFGE: pulsed-field gel electrophoresis
QRDR: quinolone-resistance-determining region
SLV: single-locus variant
ST: sequence type
