# Supplementary figures and images for: Shifts in the Antibiotic Susceptibility, Serogroups, and Clonal Complexes of Neisseria meningitidis in Shanghai, China: A Time Trend Analysis of the Pre-Quinolone and Quinolone Eras
Source: PLoS Med. 2015 Jun 9;12(6):e1001838. doi: 10.1371/journal.pmed.1001838 (PMC4461234; doi:10.1371/journal.pmed.1001838)

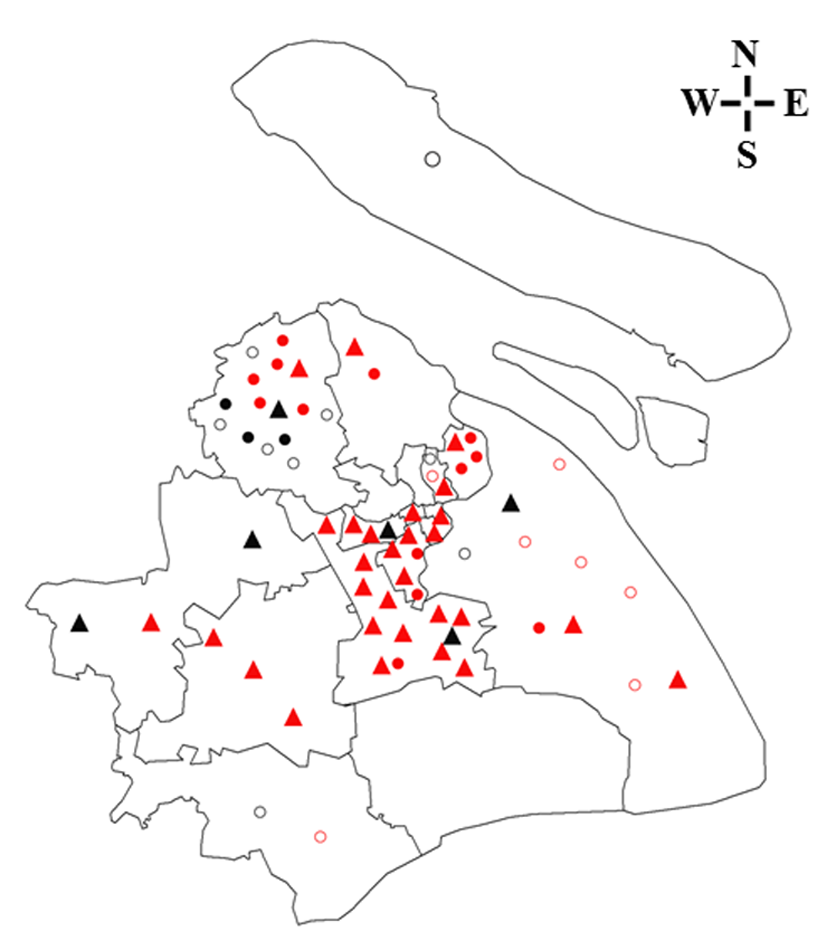

Supplement: S1 Fig — Ciprofloxacin-nonsusceptible isolates are indicated in red, including 47 ciprofloxacin-resistant isolates and four ciprofloxacin-intermediate isolates. Ciprofloxacin-susceptible isolates are indicated in black. ▲, patient; ●, close contact; ○, asymptomatic carrier. (TIF) [file pmed.1001838.s002.tif]

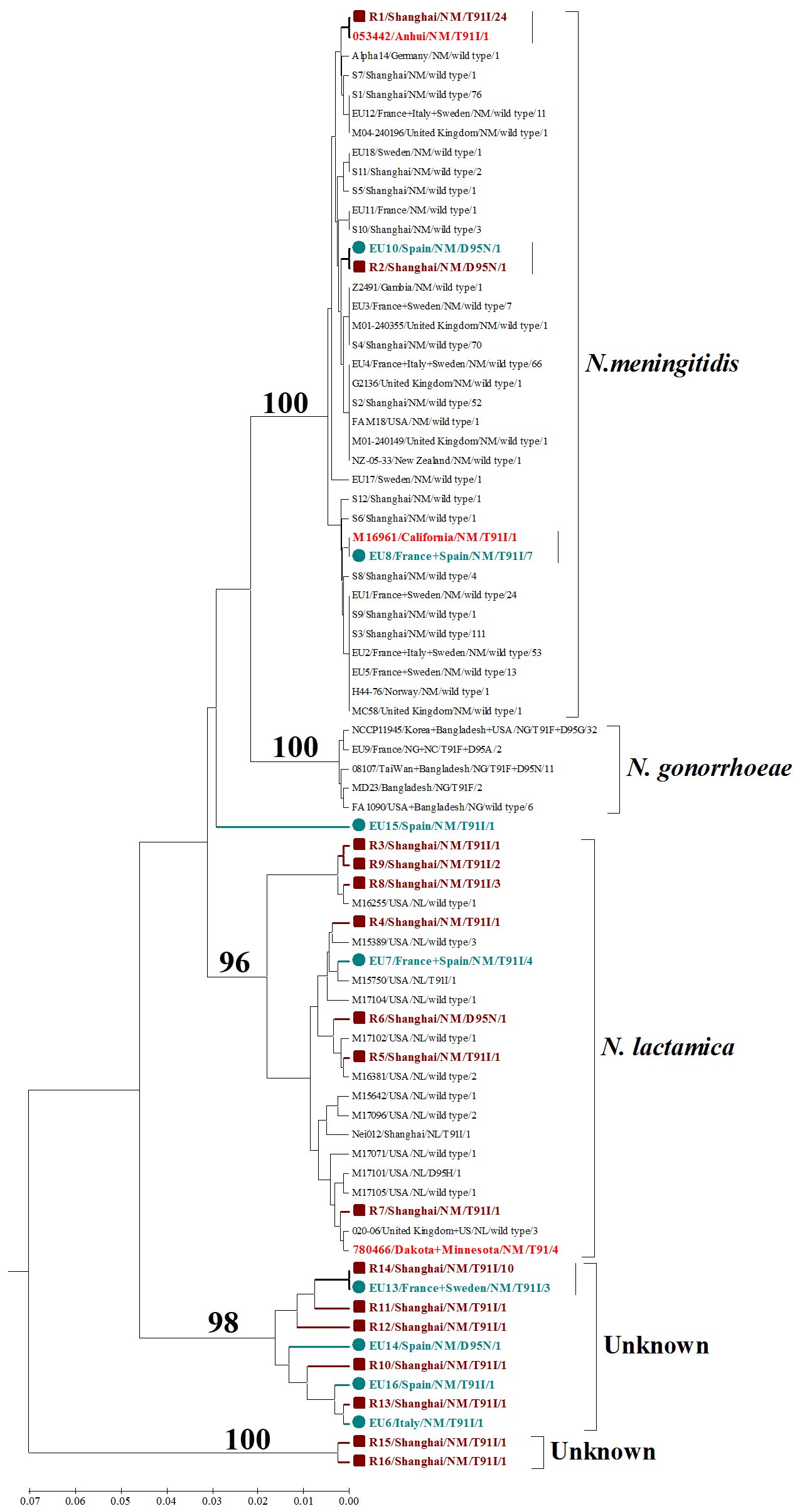

Supplement: S2 Fig — Phylogenetic analysis of the QRDR of gyrA (nucleotides 115–518) from 586 N. meningitidis (NM), 52 N. gonorrhoeae (NG), 19 N. lactamica (NL), and one N. cinerea (NC) isolates from GenBank or the Neisseria PubMLST database was conducted in MEGA 5 using the unweighted pair group method with arithmetic mean averages (UPGMA). The percentages of replicate trees in which the associated taxa clustered together in the bootstrap test (2,000 replicates) are shown next to the main branches. The clusters were determined with the bootstrap values >70% [48]. Strains are shown as “strain number or gyrA allele/country or district/species/GyrA mutation or wild type/total number.” Strains with no alteration in GyrA were assumed to be wild type. The alleles labeled with wine-colored squares were defined among ciprofloxacin-nonsusceptible isolates from Shanghai, and those labeled with cyan circles were defined by Eva Hong et al. among ciprofloxacin-nonsusceptible N. meningitidis isolates from Europe [9]. Three species clusters are indicated. (TIF) [file pmed.1001838.s003.tif]

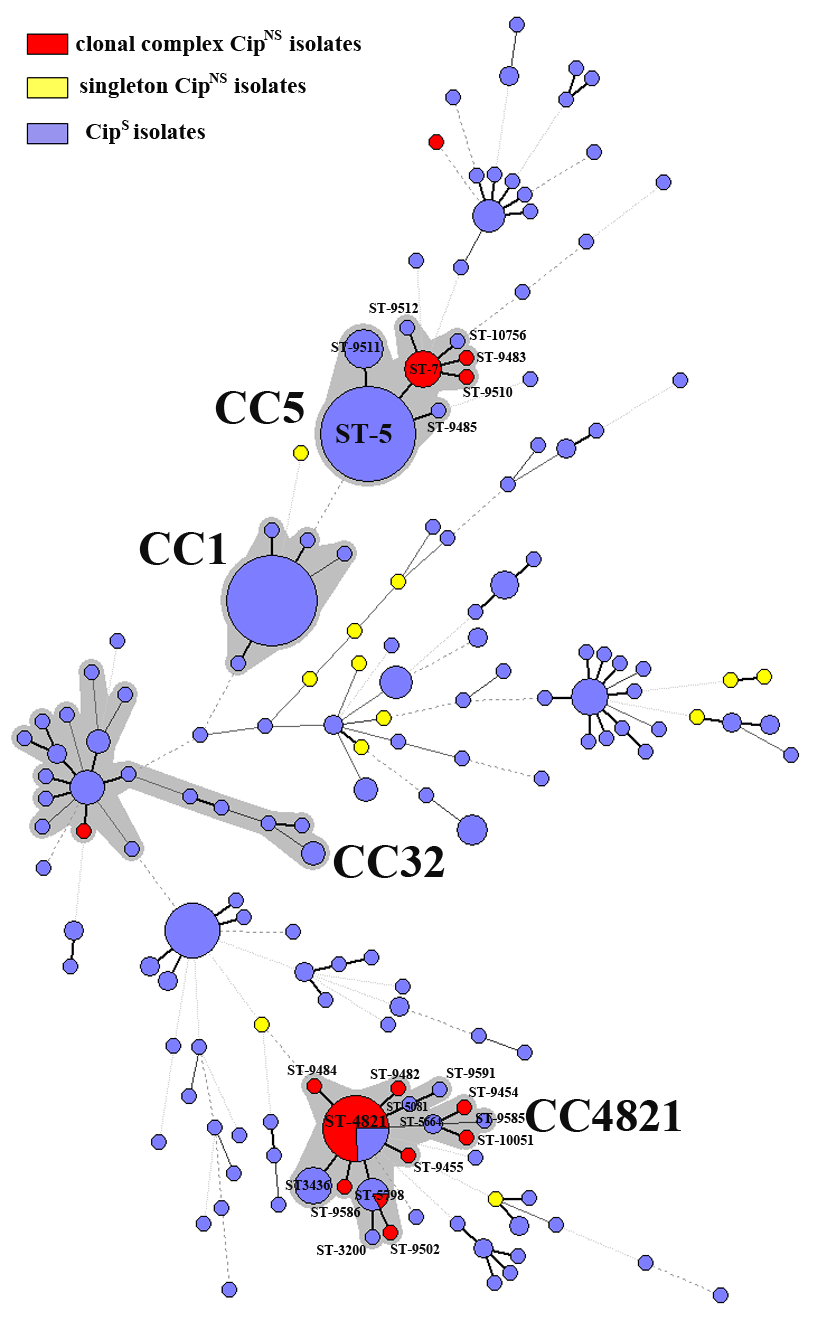

Supplement: S3 Fig — In the minimum spanning tree, the STs are displayed as circles. The size of each circle reflects the number of isolates within this particular type. The susceptibility to ciprofloxacin is represented by different colors. The colored halos surrounding the STs denote types that belong to the same CC. Heavy solid lines represent SLVs, light solid lines represent DLVs, heavy dotted lines represent triple-locus variants, and light dotted lines represent quadruple-locus variants. (TIF) [file pmed.1001838.s004.tif]
