# Supplementary material for: Shifts in the Antibiotic Susceptibility, Serogroups, and Clonal Complexes of Neisseria meningitidis in Shanghai, China: A Time Trend Analysis of the Pre-Quinolone and Quinolone Eras
Source: PLoS Med. 2015 Jun 9;12(6):e1001838. doi: 10.1371/journal.pmed.1001838 (PMC4461234; doi:10.1371/journal.pmed.1001838)
Supplement: S1 Table — (DOCX) [file pmed.1001838.s005.docx]

**S1 Table. Published CC5 ciprofloxacin-resistant *N. meningitidis* strains outside China**

| **Location** | **Year** | **No. of strains** | **Sero-**  **group** | **Sequence type** | **PorA subtype** | ***gyrA* allele**^a^ | **Ciprofloxacin  MIC(μg/ml)** | **GyrA  alteration** | **Reference** |
| --- | --- | --- | --- | --- | --- | --- | --- | --- | --- |
| France | 2004, 2006 | 2 | A | ST-7, ST-4789 ^b^ | P1.20,9 | EU13 (R14), EU8 | 0.125–0.25 | T91I | [1] |
| Spain | 2006 | 1 | Y | ST-4789 | ND^c^ | EU7 | 0.25 | T91I | [2,3] |
| Israel | 2006 | 1 | A | ST-4789 | ND | ND | 0.125 | T91I | [4] |
| Sweden | 2009 | 2 | A | ST-7, ST-8762^b^ | P1.20,9 | EU13 (R14) | 0.125–0.25 | T91I | [5] |
| Italy | 2009 | 1 | A | ST-4789 | P1.20,9 | EU6 | 0.25 | T91I | [5] |
| United Kingdom | 2011 | 1 | A | ST-4789 | P1.20,9 | EU6 | 0.19 | T91I | [5] |

^a^ These *gyrA* alleles were defined by Eva Hong *et al* [2] , and EU13 is identical to R14 which was defined in this study.

^b^ SLV of ST-7;

^c^ ND, not determined

**References:**

1. Skoczynska A, Alonso JM, Taha MK. Ciprofloxacin resistance in *Neisseria meningitidis*, France. Emerg Infect Dis. 2008; 14: 1322-1323.

2. Enriquez R, Abad R, Salcedo C, Perez S, Vazquez JA. Fluoroquinolone resistance in *Neisseria meningitidis* in Spain. J Antimicrob Chemother. 2008; 61: 286-290.

3. Alcala B, Salcedo C, de la Fuente L, Arreaza L, Uria MJ, Abad R, et al. *Neisseria meningitidis* showing decreased susceptibility to ciprofloxacin: first report in Spain. J Antimicrob Chemother. 2004; 53: 409.

4. Strahilevitz J, Adler A, Smollan G, Temper V, Keller N, Block C. Serogroup A *Neisseria meningitidis* with reduced susceptibility to ciprofloxacin. Emerg Infect Dis. 2008; 14: 1667-1669.

5. *Neisseria* multi locus sequence typing website. http://pubmlst.org/neisseria/.(accessed October 21st, 2014)
