# Supplementary material for: Shifts in the Antibiotic Susceptibility, Serogroups, and Clonal Complexes of Neisseria meningitidis in Shanghai, China: A Time Trend Analysis of the Pre-Quinolone and Quinolone Eras
Source: PLoS Med. 2015 Jun 9;12(6):e1001838. doi: 10.1371/journal.pmed.1001838 (PMC4461234; doi:10.1371/journal.pmed.1001838)
Supplement: S2 Table — (DOCX) [file pmed.1001838.s006.docx]

**S2 Table. Analysis of 13 surveys of meningococcal carriage in Shanghai from 1965 to 2013**

| **Year** | **Samples** | **Isolates (carriage rate, %)** | **Rate ratio^a^  (95% CI)** | ***p-*Value**^b^ | **Isolates tested for serogroup** | **Serogroup (%)** | | | | |
| --- | --- | --- | --- | --- | --- | --- | --- | --- | --- | --- |
|  |  |  |  |  |  | **A** | ***p-*Value^c^** | **B** | **C** | **Other than A, B and C** |
| 1966 | 249 | 39 (16) | 1 | ND^d^ | 13 | 10 (76.9) | ND | 1 (7.7) | 1 (7.7) | 1 (7.7) |
| 1967 | 5590 | 1347 (24) | 1.7 (1.24-2.35) | <0.001 | 259 | 188 (72.6) | 0.47 | 44 (17.0) | 21 (8.1) | 6 (2.3) |
| 1970 | 818 | 86 (11) | 0.6 (0.43-0.92) | 0.02 | 12 | 6 (50) | 0.02 | 0 | 6 (50) | 0 |
| 1971 | 897 | 77 (9) | 0.5 (0.35-0.75) | <0.001 | 9 | 5 (55.6) | 0.07 | 0 | 4 (44.4) | 0 |
| 1972 | 4639 | 111 (2) | 0.1 (0.09-0.19) | <0.001 | 71 | 10 (14.1) | <0.001 | 43 (60.6) | 18 (25.3) | 0 |
| 1973 | 2025 | 69 (3) | 0.2 (0.13-0.28) | <0.001 | 22 | 3 (13.6) | <0.001 | 19 (86.4) | 0 | 0 |
| 1974 | 1299 | 68 (5) | 0.3 (0.20-0.44) | <0.001 | 0 | 0 | ND | 0 | 0 | 0 |
| 1975 | 855 | 43 (5) | 0.3 (0.18-0.44) | <0.001 | 10 | 0 | <0.001 | 10 (100) | 0 | 0 |
| 1976 | 779 | 52 (7) | 0.4 (0.25-0.59) | <0.001 | 35 | 0 | <0.001 | 29 (82.9) | 6 (17.1) | 0 |
| 1977 | 3561 | 289 (8) | 0.5 (0.34-0.66) | <0.001 | 223 | 3 (1.3) | <0.001 | 196 (87.9) | 18 (8.1) | 6 (2.7) |
| 1978 | 2326 | 221 (10) | 0.6 (0.41-0.80) | 0.002 | 184 | 1 (0.5) | <0.001 | 146 (79.3) | 11 (6.0) | 26 (14.1) |
| 2007 | 553 | 11 (2) | 0.1 (0.05-0.21) | <0.001 | 11 | 0 | <0.001 | 11 (100) | 0 | 0 |
| 2010 | 644 | 4 (0.6) | 0.03 (0.01-0.09) | <0.001 | 4 | 0 | <0.001 | 2 (50) | 0 | 2 (50) |

^a^ The reference year is 1966.

^b^ Chi-squared test was used to analyze the disparity of carriage rates compared with that in 1966.

^c^ Chi-squared test was used to analyze the disparity of serogroup A proportion compared with that in 1966.

^d^ not determined.
